# Climate Change and Health Risk Perceptions of Arkansas Small Farmers through the Application of the Health Belief Model

**DOI:** 10.3390/ijerph21070955

**Published:** 2024-07-22

**Authors:** Rachel B. Hale, Keneshia Bryant-Moore, Anna Eichenberger

**Affiliations:** 1 Department of Environmental Health Sciences, College of Public Health, University of Arkansas for Medical Sciences, Little Rock, AR 72205, USA; 2 Department of Health Behavior and Education, College of Public Health, University of Arkansas for Medical Sciences, Little Rock, AR 72205, USA; kjbryant@uams.edu; 3 STEAD Scholars Program, Arkansas Department of Health, Little Rock, AR 72205, USA; eichenbergerag@hendrix.edu

**Keywords:** climate change, farmers, health, risk perceptions, health belief model

## Abstract

Climate change poses health risks to Arkansas small farmers. Farmers face an increased risk of heat-related illnesses (e.g., heat exhaustion, cerebral vascular accidents, and cardiovascular issues) and work-related injuries, death, and mental health conditions due to climate change. This cross-sectional survey employed the health belief model (HBM) as its theoretical framework. This study aimed to assess the health status of small farmers, climate change beliefs, adaptive agricultural practices, and the perceived effects of climate change on health. Study data were collected using non-probability sampling methods from small farmers (*n* = 72) with a gross farm income of < USD 250,000. The study findings show that 93% of participants reported good–excellent health, 69% believe the climate is changing and getting warmer, 58.3% believe people are responsible for the changes in our climate, and 75% believe the changing climate impacts farmers. Among the HBM predictive variables, participants reported self-efficacy (50%), perceived susceptibility (48.6%), and perceived severity (43%). Only 16.7% of farmers reported believing they have all the information needed to prepare for climate-related health impacts. This study suggests small farmers have protective factors and adaptive capacity, including health status, income, and education levels, but believe they lack the information necessary to protect their health from climate change.

## 1. Introduction

Climate change threatens the health and livelihood of small farmers globally. Within the United States (US), small farmers, defined as having an annual gross farm income under USD 250,000, face increasing risks of heat-related illnesses (such as heat exhaustion), cardiovascular diseases or conditions, and work-related injuries and death due to increasing ambient temperatures and precipitation changes due to climate change [1,2,3,4,5,6,7,8]. Small farmers are one of the populations most susceptible to experiencing the effects of climate change as their exposure is longer and with higher intensities.

Climate change interacts with social determinants of health by exacerbating their significant role in affecting farmer health [9]. Within the small farmer population, there are communities with heightened vulnerability due to existing racial and ethnic health disparities, environmental health disparities, and gender inequity [9,10]. Not only does climate change affect the physical health of farmers, but their emotional well-being is also impacted as they face economic hardships due to decreasing crop yields and workable hours. Small farmers have reported increases in anxiety and depression around climate change and the impact it will have on their livelihoods [2,3,5]. US agricultural producers rank sixth in suicide compared with other occupations, highlighting a current vulnerability climate change may exacerbate [11].

The Intergovernmental Panel on Climate Change (IPCC) defines vulnerability as “the propensity or predisposition to be adversely affected and encompasses a variety of concepts and elements including sensitivity or susceptibility to harm and lack of capacity to cope and adapt” [12]. Vulnerability is assessed by looking at the intersection between exposure and sensitivity to a threat, mediated by the capacity to adapt [12]. It aims to understand the variables that are either barriers or facilitators to building resiliency to protect their health against climate change.

It is predicted that if mitigation efforts are not enacted, the average number of unworkable hours will double by the mid-century [4]. The loss of agricultural production due to climate change may lead to higher food insecurity and poverty among small farmers and their communities [13]. US small farmers are critical for providing local communities access to a diverse and resilient food supply. Small farms serve as centers for agricultural innovation, foster community relationships, and promote agricultural practices that benefit both people and the environment [14]. Improving and protecting the health of small farmers is vital for community and environmental health.

The current literature includes studies conducted in the US focused on exploring the climate change beliefs of farmers with their motivation and willingness to adapt, without exploring how climate change may impact their health [15,16,17,18,19]. The US small farmer population, particularly in the Southeast region, is understudied, and little is known about their climate change beliefs and health risk perceptions. Risk perceptions in the context of climate change and tipping points in farming systems are crucial as they dictate how farmers perceive and react to potential disruptions that could induce significant and possibly irreversible shifts in their ecosystems or agricultural practices. Tipping points denote critical thresholds where minor alterations can trigger substantial changes [20].

The average Arkansas farmer is 57 years old and manages a 308-acre farm with yearly farm expenses of USD 124,324 and a revenue of USD 160,270 [21]. Small farmers are a unique population as they may have increased exposure and sensitivity to the effects of climate change on their health due to socioeconomic factors increasing their vulnerability [22].

A cross-national study of climate change beliefs and risk perceptions among farmers in high-income countries revealed a widespread acknowledgment of climate change, although without an association with anthropogenic causes. This trend was consistent across various locations, including the US. However, researchers noted comparatively lower belief levels among farmers when compared with the general population, with fewer farmers perceiving climate change as a significant threat to agriculture [18]. Subsequent studies in California and Puerto Rico provided further insights. In California, while a majority of farmers recognized the risks posed by climate change to global agriculture, opinions on the increase in global temperatures and the anthropogenic drivers of climate change were more divided [23]. In contrast, a study in Puerto Rico following Hurricane Maria found a majority of farmers attributed climate change to human activities [24]. These findings underscore the variability in climate change beliefs within farming communities, reflecting diverse regional perspectives and contextual factors. Such diversity highlights the complexity inherent in addressing climate change adaptation and mitigation strategies within agricultural sectors across various geographical and socio-economic landscapes.

Arkansas farmers are already feeling the effects of climate change, including major flooding resulting in millions of damages alongside a record drought in 2022 affecting barge access on the Mississippi River [7,25,26]. This quantitative study aimed to assess the current health status of Arkansas small farmers and current adaptative agricultural practices and to identify the perceived relationship between climate change and personal health impacts on Arkansas small farmers based on the constructs of the health belief model. The HBM is a theoretical framework that helps explain and predict health behaviors by focusing on individuals’ beliefs about health conditions. The HBM is useful for assessing a small farmer’s perceptions about how climate change may impact their health because it can identify the farmer’s beliefs about susceptibility to climate-related health risks, the severity of these risks, the benefits of taking adaptive measures, and the barriers to implementing these measures. Understanding these beliefs can help in designing effective interventions and communication strategies that motivate farmers to undertake actions to protect their health against the impacts of climate change [27].

## 2. Materials and Methods

**Figure 1:**
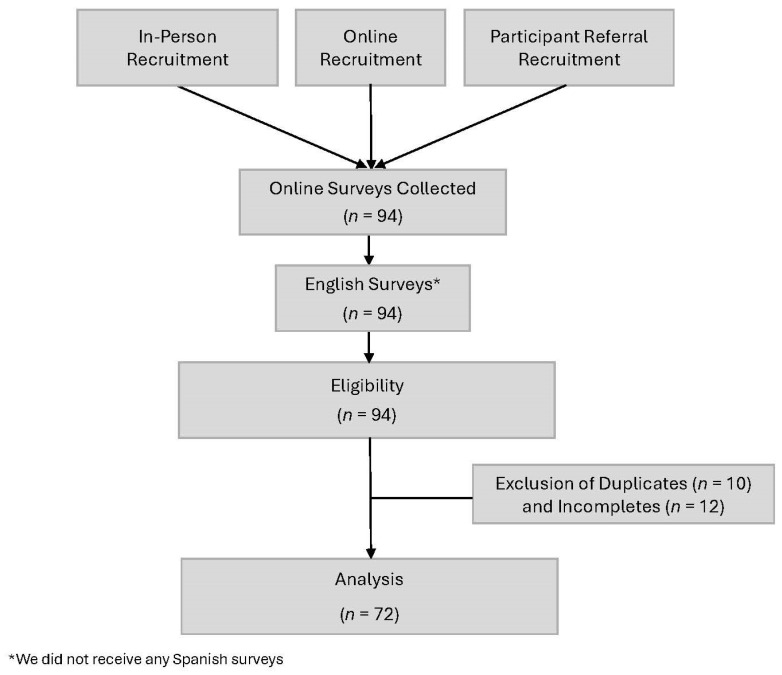
Project flow chart.

This descriptive cross-sectional survey employed a questionnaire collecting demographics, health status, agricultural practices, climate change beliefs, and climate change and health risk perceptions (Figure 1). The study protocol was approved by the University of Arkansas for Medical Sciences (UAMS) Institutional Review Board.

The study population included small farmers throughout Arkansas. Approximately 97% of Arkansas farms are family-owned, and agriculture is the state’s largest industry, covering from specialty crops to row crops such as rice, soybeans, and cotton. The inclusion criteria required participants to be (i) 18 years or older, (ii) owners or co-owners of a small farm in Arkansas with a gross cash farm income under USD 250,000, (iii) Arkansas residents, and (iv) English and/or Spanish speakers. This project utilized the Total Worker Health© (TWH) approach by aligning the study population with the defining element of TWH, leadership commitment, as small farmers are the primary decision-makers and leaders in their operations [28,29].

Non-probability sampling methods, such as convenience, voluntary response, and snowball sampling, were utilized with primary recruitment conducted through social media, in-person recruitment at farmers’ markets, email blasts from community partners such as the Arkansas Local Food Network and the University of Arkansas Cooperative Extension Office, and physical flyers posted at agricultural stores.

The English and Spanish surveys were provided to participants in three formats: in-person, electronically, and on paper. However, all participants chose to complete the survey online via Research Electronic Data Capture (REDCap). REDCap was developed in 2004 by Vanderbilt University and is a free, secure web-based application for building and managing online databases and surveys for research studies. The research team worked with the Translational Research Institute (TRI) Community Engagement team and their community partners, Arkansas Interfaith Power and Light, and the Arkansas Local Food Network, to disseminate the survey to their small farmers. In addition, members of the research team visited local farmers’ markets across the state, including small roadside stands. Survey participants were provided a USD 20.00 gift card. Due to the invasion of bots, much of the survey collection was conducted via direct messaging on social media rather than public posts and via email.

The 42-question survey (Appendix A) was designed by adapting questions from the BRFFS 2021 Survey and the Tobacco, Alcohol, Medication, and Other Substances Tool (TAPS), both validated instruments, alongside questions adapted from existing climate change and health belief instruments [30,31]. The instrument included demographic and farm characteristic questions. To combat participant hesitancy due to polarizing terminology such as “climate change” and “global warming”, the survey employed the phrase “our changing climate” when applicable [32]. The median time to complete the survey was 15 min.

The HBM constructs were used to develop the final questions regarding climate change, climate change and health beliefs, and risk perceptions. All perception questions used a variety of Likert-type responses such as strongly agree to strongly disagree. 

To ensure simplicity in the language and that the length was appropriate, the instrument was reviewed by community partners who were either small farmers or worked directly with the farming community. In addition, the survey materials were reviewed by the UAMS Center for Health Literacy (CHL) for plain language assessment and Spanish translation, which included readability assessments using multiple validated formulas, to estimate the level of difficulty of the reading material. In addition to a formal evaluation of how easy the content was to understand and act upon, the Patient Education Materials Assessment Tool (PEMAT) was used [33,34,35]. The PEMAT is a standardized process in which two or more team members follow a protocol for independent review and then discuss to arrive at a consensus. After formally assessing the documents, the CHL’s team collaboratively edits each project toward a difficulty level of “easy” (less than 7th grade) or better and content that is organized, understandable, and actionable.

Prior to analysis, surveys were reviewed for completion in REDCap. We excluded 12 incomplete surveys and 10 duplicate entries. Data were entered into the Statistical Package for Social Sciences, SPSS (v18) for Windows, and descriptive analyses were conducted with unweighted data. Simple descriptive statistics tools such as frequency and percentage were used to describe the sociodemographic and health characteristics of the participants.

## 3. Results

**Figure 2:**
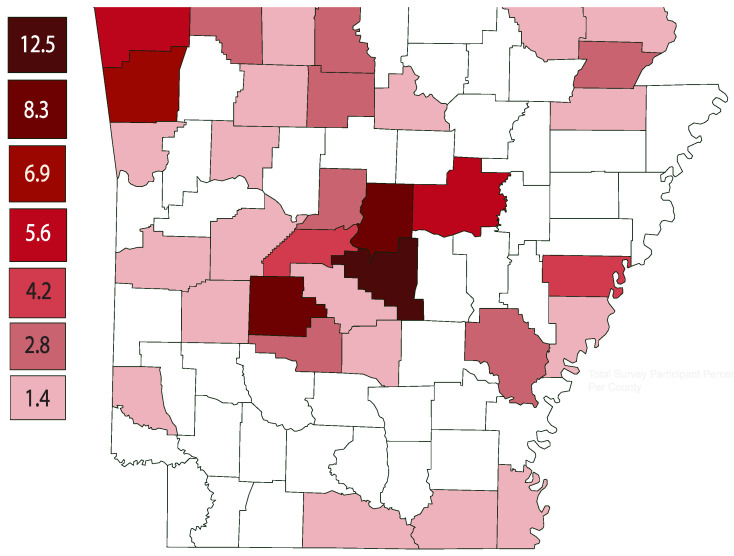
Counties represented in the survey.

A total of 72 Arkansas small farmers completed the survey between June 2023 and December 2023. The majority of the participants were White (81.0%), female (61.1%), and between the ages of 35 and 54 years old (51.4%). Over 65% of the participants reported having a bachelor’s degree or higher and 66.7% had a household income of USD 75,000 or higher. The majority of the participants reported farming was not their primary job (68.1%), 93.1% selling what they produce and/or raise, and 46.0% of the participants reported farming for five years or less (Table 1). Figure 2 illustrates the counties represented in the survey indicating high participation by small farmers in Central and Northwest Arkansas.

The majority reported good to excellent overall health (93%), with almost all having health insurance (98.6%). The participants reported the highest health problems as arthritis pain/swelling joints (29.2%), hypertension (26.4%), high cholesterol (25%), and depressive disorder (22.2%).

Regarding substance use, most participants reported they did not use tobacco products (91.7%) or any illegal drugs (87.5%). Less than half (34.7%) reported that they had not consumed any alcohol in the past year, and the majority (94.4%) reported not taking any unprescribed medications.

### 3.1. Current Adaptative Agricultural Practices

Small farmers reported the implementation of crop rotation (37.5%), manure/compost (44.4%), drip irrigation (33.3%), hoop houses (29.2%), and managing livestock on perennial and annual forage rotation (27.8%) to a great extent. The majority of participants reported no use of solar panels (80.4%), agrisolar (91.7%), or hydroponics (83.3%).

### 3.2. Perceived Relationship between Climate Change and Personal Health Impacts on Arkansas Small Farmers

The small farmers believed the climate is changing and getting warmer (69.5%), people are responsible for the changes in the climate (58.4%), and this impacts farmers (75%). Over half (59.7%) of the participants believed the changing climate is having a negative impact on Arkansas agriculture and impacting their ability to grow crops (Table 2). Table 3 illustrates the climate change and health belief risk perceptions of the participants. The survey statements were based on the Health Belief Model. Among the predictive variables of the HBM, participants reported self-efficacy (50%), perceived susceptibility (48.6%), and perceived severity (43%). Only 16.7% of farmers reported they believe they have all the information needed to prepare for the health impacts of climate change (Table 3).

## 4. Discussion

This study, to the best of our knowledge, is the first quantitative inquiry of Arkansas small farmers about their risk perceptions and beliefs about climate change, specifically framing the questions within the health lens. As much of the existing literature on North American farmers does not include inquiries about individual beliefs about climate change impacts on their personal health, our findings provide new insights into practical implications that can guide efforts to improve adaptation and mitigation adoption in Arkansas and the broader southeastern region of the United States.

The study results were surprising based on 2023 Yale Opinion Maps from Arkansas residents concerning their beliefs about climate change [36]. Compared with Arkansas residents, small farmers have a higher belief in anthropogenic climate change, and they and their livelihoods will be negatively affected by climate change. Unlike the results of small farmers in New York [15] and in Michigan [37], Arkansas small farmers appear to have a higher belief in climate change, with those who do not believe in climate change being the minority of participants. Future qualitative studies may inquire into what is driving these beliefs in comparison with average Arkansas residents.

The overall health status of the survey participants was healthier than Arkansans who reported excellent–good health (76.2%) in the 2022 BRFSS Survey [38]. This may be impacted by the higher education and income levels of small farmers. Per the 2018–2022 American Community Survey, 24.7% of Arkansas residents reported having a bachelor’s degree or higher compared with 65% of small farmer survey participants [39]. Over half of the small farmers reported a household income of USD 75,000 or higher compared with the median Arkansan household income of USD 56,335. Additionally, more than half of farmers in the United States require a second occupation to survive, including having access to health insurance [40]. We found this to be true among our survey participants as over half reported farming was not their main occupation. These factors may also indicate those who had access to the survey are a far more protected community within Arkansas from climate change compared with the general population.

Though the survey was offered to the Spanish-speaking community and targeted recruitment was conducted, the project did not receive any surveys in Spanish. This might be due to various factors, including mistrust and the overall demographics of small farmers in Arkansas. Spanish-speaking small farmers may not consider themselves to be a small farmer if they grow their produce for household consumption only. Future studies may inquire within the farmworker community in southern and eastern portions of Arkansas as this survey found many small farmers are growing specialty crops in fewer acreages predominately in central and northwest Arkansas counties. 

The implementation of adaptive agricultural practices is less focused on new technology, such as solar panels, agrisolar, or hydroponics, compared with more common practices, such as the use of manure/compost. This is similar to Oregonian farmers surveyed in 2019–2020, whose main adaptation practice focused on improving soil health [41]. Though these common practices will enable small farmers to adapt to climate change, the incorporation of technology may provide beneficial advantages for the future. US small farmers exhibit varying adaptive capacities influenced by factors such as access to financial resources, education, technology, social networks, and institutional support. Their ability to adapt to climate change and economic pressures is enhanced by knowledge exchange, the diversification of income sources, and community resilience practices. However, limited access to capital and information can hinder their adaptive responses [42].

The Health Belief Model constructs framed the climate change and health questions. As previously mentioned, almost half of the small farmers indicated they believe they can adapt to the possible risks and problems due to our changing climate (self-efficacy), with close to half acknowledging their susceptibility and the severity of health impacts from climate change. These responses may imply the existing motivation of small farmers to perform adaptative actions to protect their health. These results are similar to those of a study on Iowa farmers that found a farmer’s belief in climate change and perception of it as a problem activate their willingness to take action [19].

The survey did expose limited cues to action, which will require additional stimuli to trigger these actions. For example, small farmers may need action-driven information through health prevention education (i.e., heat illness prevention) to increase their ability to prevent and respond to health concerns. Health prevention education and other incentivized agricultural programs may increase their perceived benefits and lower their perceived barriers. Further research is required to understand the appropriate messaging within these programs to encourage farmers who do not believe in climate change to participate. For example, climate framing such as “weather extremes” and “extreme weather events” may persuade more farmer participation despite their climate change beliefs.

## 5. Conclusions

This study suggests small farmers have protective factors, including health status, income, health insurance, and education levels, which may indicate the potential for adaptive capacity. Most participants reported farming as a secondary occupation, which may have limited their exposure to extreme weather events, such as heat, to their health. The participants primarily grew specialty crops to sell and/or keep for personal use. The participants appeared to understand the severity and risk to their health due to climate change. The responses indicate they believed they lacked the necessary information to protect their health from climate change, which may be why they had lower perceived benefits and barriers. The study results can be used as the basis to explore how to disseminate information to small farmers on protecting their health from climate change. In addition, the results illustrate the current adaptative agricultural practices that can be built upon and further explored by understanding how they may mitigate the health impacts of climate change.

There are limitations to this study stemming from the challenges associated with accessing the farming community. This study’s findings may be subject to sampling bias due to the difficulty in obtaining representative samples of farmers. Farmers often have busy schedules and may be reluctant to participate in research activities, potentially leading to a non-random sample that does not fully reflect the diversity of opinions within the farming population. In addition, due to distinct differences between farming communities due to geographic location, this information may not be generalizable to other communities. Despite these limitations, efforts to access and work alongside farming communities are crucial for understanding the complex interactions between climate change perceptions, agricultural practices, and adaptation strategies.

In addition, to assess the health impacts of climate change on agricultural workers, future studies should assess mid- to large-scale farm owners alongside farmworkers. Farmworkers may have a higher duration of exposure and limited adaptative capacity due to multiple factors.

## Figures and Tables

**Table 1:** Participant characteristics among Arkansas small farmers % (n).

Variable	Category	% (n)
Age	18–24 years old	1.4 (1)
	25–34 years old	11.1 (8)
	35–44 years old	29.2 (21)
	45–54 years old	22.2 (16)
	55–64 years old	19.4 (14)
	65–74 years old	16.7 (12)
Gender	Female	61.1 (44)
	Male	38.9 (28)
Race/ethnicity	American Indian/Alaskan Native	2.7 (2)
	Asian or other Pacific Islander	1.3 (1)
	Black	8.3 (6)
	Multiracial	4.1 (3)
	White	81.0 (58)
	Other: Italian American	1.3 (1)
	Hispanic/Latino/Spanish origin	4.2 (3)
Education	Some high school	2.8 (2)
	General Education Development (GED)	1.4 (1)
	High School Diploma	13.9 (10)
	Some college	1.4 (1)
	Associate degree	15.3 (11)
	Bachelor’s degree or higher	65.3 (47)
Annual household income	<USD 35,000	13.9 (10)
	<USD 50,000	16.7 (12)
	<USD 75,000	20.8 (15)
	<USD 100,000	34.7 (25)
	<USD 250,000	5.6 (4)
	USD 250,000 or more	5.6 (4)
Years as a farmer ^1^	5 years or less	46.0 (33)
	6–14 years	29.2 (21)
	15–22 years	12.5 (9)
	25 years or more	9.72 (7)
Farming is their primary job	Yes	31.9 (23)
	No	68.1 (49)
Sell what they produce/raise	Yes	93.1 (67)
	No	6.9 (5)

^1^ Missing 2 values.

**Table 2:** Climate change beliefs.

Survey Statements	Category	% (n)
Our climate is changing and getting warmer	Strongly Disagree–Disagree	9.7 (7)
	Neither Agree or Disagree	20.8 (15)
	Agree–Strongly Agree	69.5 (50)
People are responsible for the changes in our climate.	Strongly Disagree–Disagree	20.8 (15)
	Neither Agree or Disagree	20.8 (15)
	Agree–Strongly Agree	58.4 (42)
Our changing climate is having a negative impact on agriculture in Arkansas.	Strongly Disagree–Disagree	13.9 (10)
	Neither Agree or Disagree	26.4 (19)
	Agree–Strongly Agree	59.7 (43)
Our changing climate is impacting a farmer’s ability to grow crops.	Strongly Disagree–Disagree	12.5 (9)
	Neither Agree or Disagree	27.8 (20)
	Agree–Strongly Agree	59.7 (43)
Our changing climate will affect future generations, but it does not affect the current generation.	Strongly Disagree–Disagree	52.7 (38)
	Neither Agree or Disagree	27.8 (20)
	Agree–Strongly Agree	19.5 (14)
Our changing climate impacts farmers.	Strongly Disagree–Disagree	9.7 (7)
	Neither Agree or Disagree	15.3 (11)
	Agree–Strongly Agree	75 (54)

**Table 3:** Climate change and health belief model (HBM) Constructs.

HBM Construct	Survey Statements	Category	% (n)
Perceived susceptibility	Our changing climate will affect my personal health.	Strongly Disagree–Disagree	13.9 (10)
		Neither Agree or Disagree	37.5 (27)
		Agree–Strongly Agree	48.6 (35)
Perceived severity	Our changing climate can put my life and livelihood at risk.	Strongly Disagree–Disagree	18.0 (13)
		Neither Agree or Disagree	39.0 (28)
		Agree–Strongly Agree	43.0 (31)
Perceived benefits	If I change my farm practices, I can protect my health from our changing climate.	Strongly Disagree–Disagree	27.8 (20)
		Neither Agree or Disagree	37.5 (27)
		Agree–Strongly Agree	34.8 (25)
Perceived barriers	I cannot protect myself from the harmful impacts of our changing climate.	Strongly Disagree–Disagree	34.7 (25)
		Neither Agree or Disagree	38.9 (28)
		Agree–Strongly Agree	26.6 (19)
Cues to action	I have all the information to prepare for the impacts of our changing climate on my health.	Strongly Disagree–Disagree	36.1 (26)
		Neither Agree or Disagree	47.2 (34)
		Agree–Strongly Agree	16.7 (12)
Perceived self-efficacy	I can adapt to the possible risks and problems from our changing climate.	Strongly Disagree–Disagree	13.8 (10)
		Neither Agree or Disagree	36.1 (26)
		Agree–Strongly Agree	50.0 (36)

## Data Availability

The anonymized data of this study may be provided upon request to the first author.
